# COVID-19 Burden on HIV Patients Attending Antiretroviral Therapy in Addis Ababa, Ethiopia: A Multicenter Cross-Sectional Study

**DOI:** 10.3389/fmed.2022.741862

**Published:** 2022-03-02

**Authors:** Dagmawi Chilot, Yimtubezinash Woldeamanuel, Tsegahun Manyazewal

**Affiliations:** ^1^Center for Innovative Drug Development and Therapeutic Trials for Africa (CDT-Africa), College of Health Sciences, Addis Ababa University, Addis Ababa, Ethiopia; ^2^College of Medicine and Health Sciences, University of Gondar, Gondar, Ethiopia

**Keywords:** coronavirus disease 2019 (COVID-19), severe acute respiratory syndrome coronavirus 2 (SARS-CoV-2), HIV, clinical care, treatment, antiretroviral therapy, Ethiopia

## Abstract

**Background:**

There has been promising progress toward screening, testing, and retaining patients with HIV in care in Ethiopia. Concern exists that possible disruptions in HIV programs due to coronavirus disease 2019 (COVID-19) could result in a more HIV-related mortality and new HIV infections. This study aimed to investigate the real-time burden of COVID-19 on patients with HIV attending antiretroviral therapy.

**Methods:**

We conducted a facility-based, multicenter, and cross-sectional study among patients with HIV attending antiretroviral therapy in 10 healthcare facilities in Addis Ababa, Ethiopia, in the COVID-19 pandemic period. Data were collected using adapted, interviewer-based questionnaires, and entered into EpiInfo version 7 and exported to SPSS version 26 for analysis.

**Result:**

A total of 212 patients with HIV were included. The participants who missed visits for refill were 58 (27.4%). When the effects of other independent variables on appointments/visits for refill were controlled, the following characteristics were found to be the most important predictors of missed appointments (*p*< *0.05*): age ≥ 55 [adjusted odds ratio (AOR) = 6.73, 95% CI (1.495–30.310)], fear of COVID-19 [AOR = 24.93, 95% CI (2.798–222.279)], transport disruption [AOR = 4.90, 95% CI (1.031–23.174)], reduced income for traveling to a health facility [AOR = 5.64, 95% CI (1.234-25.812)], limited access to masks [AOR = 7.67, 95% CI (1.303–45.174)], sanitizer [AOR = 0.07, 95% CI (0.007–0.729)], and non-medical support [AOR = 2.32, 95% CI (1.547–12.596)]. The participants were well aware of the COVID-19 preventive measures. The most costly COVID-19 preventive measures that cause financial burden to the patients were the costs for buying face masks (63.7%), disinfectants (55.2%), and soap for handwashing (22.2%). The participants who missed follow-up diagnostic tests were 56 (26.4%). Variables, which were found to be statistically significant, include the following: age ≥ 55 [AOR = 0.22, 95% CI (0.076–0.621)], partial lockdown [AOR = 0.10, 95% CI (0.011–0.833)], limited access to health services [AOR = 0.15, 95% CI (0.045–0.475)], reduced income for traveling to health facility [AOR = 0.18, 95% CI (0.039–0.784)], and unable to get masks [AOR = 0.12, 95% CI (0.026–0.543)]. The participants who missed counseling services were 55 (25.9%). In multivariate logistic regression, the following were statistically significant: age ≥ 55 [AOR = 0.21, 95% CI (0.078–0.570)], fear of COVID-19 [AOR = 0.11, 95% CI (0.013–0.912)], reduced income [AOR = 0.17, 95% CI (0.041–0.699)], unable to get face masks [AOR = 0.19, 95% CI (0.039–0.959)], and partial lockdown [AOR = 0.08, 95% CI (0.008–0.790)].

**Conclusions:**

The COVID-19 had a significant burden on patients with HIV to attend their routine clinical care and treatment, which may lead to treatment failure and drug resistance. The impact was on their appointments for medication refills and clinical and laboratory follow-ups. Targeted initiatives are needed to sustain HIV clinical care and treatment services and improve the well-being of people living with HIV.

## Background

Coronavirus disease 2019 (COVID-19) could be the most catastrophic pandemic in modern history. It has infected over 173,674,509 people globally and resulted in more than 3,744,408 deaths as of June 9, 2021 (1). Countries have been taking strong preventive measures to reduce and curve the transmission (2–4). Many health care professionals shifted and health facilities were repurposed into targeted COVID-19 centers to manage patients (5–7). Evidence showed these measures have led to restrictions of health facilities to the management of emergency medical conditions and chronic diseases care and treatment services (8, 9). Ethiopia, a country in sub-Saharan Africa (SSA), is categorized under COVID-19 epidemic phase III (advancing outbreak) according to the Africa Centers for Disease Control and Prevention (Africa CDC) classification (10). On March 13, 2020, the first known case of COVID-19 in Ethiopia was reported in the capital city (11). As of December 17, 2021, people that have been diagnosed with the coronavirus were 374,402, of whom 6,855 (1.83%) died, 16,850 (4.50%) are still sick, and 350,697 (93.67%) have recovered (12). Addis Ababa became the first major city in Ethiopia to report cases and deaths from COVID-19. In Ethiopia, COVID-19 imposed a burden on physical infrastructure and exacerbated the preexisting weaknesses of health systems. As the country has limited numbers of hospitals and health centers, it presented a significant challenge to manage the pandemic and other diseases simultaneously (11–13).

By the end of 2020, it was estimated that 37.6 million people have HIV infection globally, and 1.5 million are newly infected. Only 27.4 million of them are on treatment with antiretroviral therapy (ART), which means 10.2 million (27%) people are still remains untreated with ART (14). The HIV remains highly prevalent in Africa, accounting for more than 67% of the people living with HIV/AIDS (PLWH) worldwide (15). The sub-Saharan region is the most affected place in the world with 25.6 million PLWH (16). Ethiopia is one of the majorly affected countries in sub-Saharan Africa with a national prevalence rate 0.9% (17). Concern exists that possible disruptions in HIV programs due to COVID-19 could result in more HIV-related mortality and new HIV infections.

The double burden of COVID-19 and HIV is one of the major health challenges, especially in developing countries with high HIV prevalence (18). The PLWH might be particularly at high risk for infection with poor clinical outcomes (19–22). Containment measures, disruptions to supply chains, and loss of income have the potential to exacerbate the impacts of the pandemic on patients with HIV (23). While these impacts will vary significantly across countries, some recommended providing ART for 3–6 months, and others began to offer home delivery services through volunteers to reduce the adverse health outcomes (24, 25). The extensive demand for physicians has led to the rescheduling of routine reviews and hospital visits of patients with HIV (26–28). Fear of COVID-19 exacerbated food insecurity, and COVID-19 protective behaviors hindered voluntary HIV testing and healthcare services.

Many countries warned that they are at risk of stock-outs of antiretroviral (ARV) medicines, and some have critically low stocks as a result of the pandemic (29). In addition, PLWH were doubtful about the availability of ART services and about which HIV clinic to attend in the pandemic period (30).

There are limited real-time patient-level pieces of research on how effective and useful country-level COVID-19 interventions were for patients with HIV. As well, the impact of the COVID-19 pandemic on HIV at a population level is not well-known. With the limited level of evidence in the world and as to our knowledge, no research was done on the impact of the pandemic on patients attending HIV care and treatment services in Ethiopia. There is an urgent need for adequately powered studies that investigate the impact of COVID-19 on HIV clinical care and treatment to augment the health of people living with HIV.

Thus, this study aimed to investigate the real-time burden of COVID-19 on people living with HIV who were attending antiretroviral therapy facilities in Addis Ababa, Ethiopia.

## Methods

### Design and Setting

A cross-sectional multi-center study was carried out at 10 primary health care centers in Addis Ababa, from March 15 to April 25, 2021. The city has 10 sub-cities and 116 woredas, and has different government health facilities, including six hospitals and 106 public health centers. In Ethiopia, the COVID-19 pandemic is higher in the capital Addis Ababa (31). Addis Ababa is the highest in HIV prevalence next to Gambella regional state (32). The study was conducted in 10 health facilities, one in each sub-city, which has high flow of patients with HIV.

### Participants

In this study, the source population was all patients with HIV of age > 18 years attending care and treatment in the selected health centers. The study population were those who were attending care and treatment services during the data collection period. The participants were included if they were (I) the patients with HIV, as confirmed within the study facilities or result referred from another health facility; (II) a man or a woman aged ≥ 18 years; (III) volunteered to participate in the study. As this study was conducted during the high COVID-19 time in Ethiopia, we approached only 212 participants to minimize the exposure for the pandemic during the interview. All eligible participants who have been attending clinical care and treatment in those study sites during the data collection period were considered with strict precautions to prevent the transmission of the corona virus. The health facilities were selected purposively, one in each subcity, where routine HIV care and treatment services are given and provide services for large number of patients with HIV in comparison with other health centers in the subcities. Patients with HIV who attend clinical care and treatment services during the data collection period were taken from each health facility (Table 1).

**Table 1:** Sampling procedure.

**Name of the health facility**	**Sub-city**	**No. of. HIV cases on ART**
Addis raey HC	Addis ketema	20
Akaki HC	Akakikality	50
Kebena HC	Arada	18
Goro HC	Bole	11
Addisugebya HC	Gulele	16
Kazanchis HC	Kirkos	30
Alem bank	Kolfe	10
T/haymanot HC	Lideta	36
Woreda 02 HC	Nifas-silk lafto	10
Woreda 13 HC	Yeka	11

### Data Collection

The questionnaire was developed by reviewing relevant literature to ensure reliability. The questionnaire was adapted, pre-tested, and structured to collect primary data for the assessment of the overall impact of COVID-19. During data collection procedures, all the collected data were reviewed and checked daily for their completeness. The data collection instrument was developed in English and was translated to Amharic, and later back-translated to English to check for any inconsistencies or distortion in the meaning and concepts of the words by another person. Eligible participants who were attending the selected health centers were invited to participate. The participants were given information about the study through an information sheet and signed a consent form if they agreed to be part of the study. The data collectors and supervisors were trained before the actual data collection period regarding the approach, objective of the study, and ethical issues. The data collection was interviewer administered, and the questionnaire includes sections, such as sociodemographic characteristics, awareness about preventive measures, care, and treatment services.

### Data Analysis and Interpretation

All questionnaires were checked for completeness every day by the principal investigator and supervisors. Data cleaning was conducted at the end of the data entry. The analysis was done using bivariate and multivariate logistic regression to observe the effects of independent variables on the outcome variable while simultaneously controlling for other potential confounding factors. The raw data entered into Epi Info version 7 to control entry errors and exported to SPSS 26 for analysis.

## Results

### Sociodemographic Characteristics

A total of 212 patients with HIV were enrolled in the study, with a response rate of 100%, and 133 (62.7%) were female. Of the total, 103 (48.6%) were in the age group 35–54 years. Most of them (41.5%) were married, and 59 (27.8%) had attended primary education. One hundred and forty-six (68.9%) were Orthodox Christian, and 24.1% were governmental employees (Table 2).

**Table 2:** Sociodemographic characteristics of respondents, Addis Ababa, Ethiopia, May 2021.

**Variables**	**Category**	**Frequency**	**Percentage**
Sex	Male	79	37.3%
	Female	133	62.7%
Age	18–34	55	25.9%
	35–54	103	48.6%
	≥55	54	25.5%
Marital status	Single	50	23.6%
	Married	88	41.5%
	Widowed	40	18.9%
	Divorced	26	12.3%
	Separated	8	3.8%
Level of education	No education	46	21.7%
	Can read and write	30	14.2%
	Primary education	59	27.8%
	Secondary education	44	20.8%
	Diploma and above	33	15.6%
Religion	Orthodox	146	68.9%
	Muslim	36	17.0%
	Protestant	21	9.9%
	Catholic	3	1.4%
	Others	6	2.8%
Occupation	Student	3	1.4%
	Daily laborer	41	19.3%
	Merchant	22	10.4%
	Governmental employee	51	24.1%
	Private/NGO employee	43	20.3%
	Farmer	5	2.4%
	Housewife/unemployed	47	22.2%

### Most Effective Preventive Measure of COVID-19

Most participants (86.8%) responded “Cover mouth nose with a face mask” is the most effective preventive measure of COVID-19. Responses of the study participants on preventive measures such as “stay at home” and “use disinfectant” were 77.4%, 76.4%, respectively (Table 3).

**Table 3:** Awareness of respondents on COVID-19 preventive measure, Addis Ababa, Ethiopia, May 2021.

**Variables**	**Category**	**Frequency**	**Percentage**
Stay at home	No	48	22.6%
	Yes	164	77.4%
Maintain physical distancing	No	87	41.0%
	Yes	125	59.0%
Avoid close contact	No	85	40.1%
	Yes	127	59.9%
Cover mouth nose with facemask	No	28	13.2%
	Yes	184	86.8%
Frequent handwashing with soap	No	43	20.3%
	Yes	169	79.7%
Avoid touching of eyes nose and mouth with unwashed hands	No	68	32.1%
	Yes	144	67.9%
Avoid mass gathering	No	92	43.4%
	Yes	120	56.6%
Restrict movement	No	98	46.2%
	Yes	114	53.8%
Use disinfectant	No	50	23.6%
	Yes	162	76.4%

### The Financial Burden of COVID-19

The most costly COVID-19 preventive measures that cause financial burden to the patients were costs for buying face masks [135 (63.7%)], disinfectants [117 (55.2%)], and soap for handwashing [47 (22.2%)] (Table 4).

**Table 4:** Financial burden of the respondents on the COVID-19 preventive measures, Addis Ababa, Ethiopia, May 2021.

**Variables**	**Category**	**Frequency**	**Percentage**
Facemask	No	77	36.3%
	Yes	135	63.7%
Soap for frequent hand washing	No	165	77.8%
	Yes	47	22.2%
Disinfectant	No	95	44.8%
	Yes	117	55.2%

### HIV Care and Treatment Services During COVID-19

The participants who obliged to change a health center were three (1.4%), and 27 (12.7%) denied health services. Almost all the participants said health care providers were polite and respectful (99.5%), willing to listen and answer their questions (99.5%), give attention to their individual needs (99.1%) (Table 5).

**Table 5:** Response of study participants on health care facilities and service delivery, Addis Ababa, Ethiopia, May 2021.

**Variables**	**Category**	**Frequency**	**Percentage**
Obliged to change the health center because of this pandemic?	Yes	3	1.4%
	No	209	98.6%
Denied health services?	Yes	27	12.7%
	No	185	87.3%
Politeness and respect of health professionals?	Yes	211	99.5%
	No	1	0.5%
Willingness of professionals to listen and answer your questions?	Yes	211	99.5%
	No	1	0.5%
Attention of professionals to your individual needs?	Yes	210	99.1%
	No	2	0.9%
Staff seemed uncomfortable with you?	Yes	23	10.8%
	No	189	89.2%
Contact care provider when there is a health problem or comorbidities quickly?	Yes	101	47.6%
	No	111	52.4%

### Main Barriers to Access Health Care During the Pandemic

**Figure 1:**
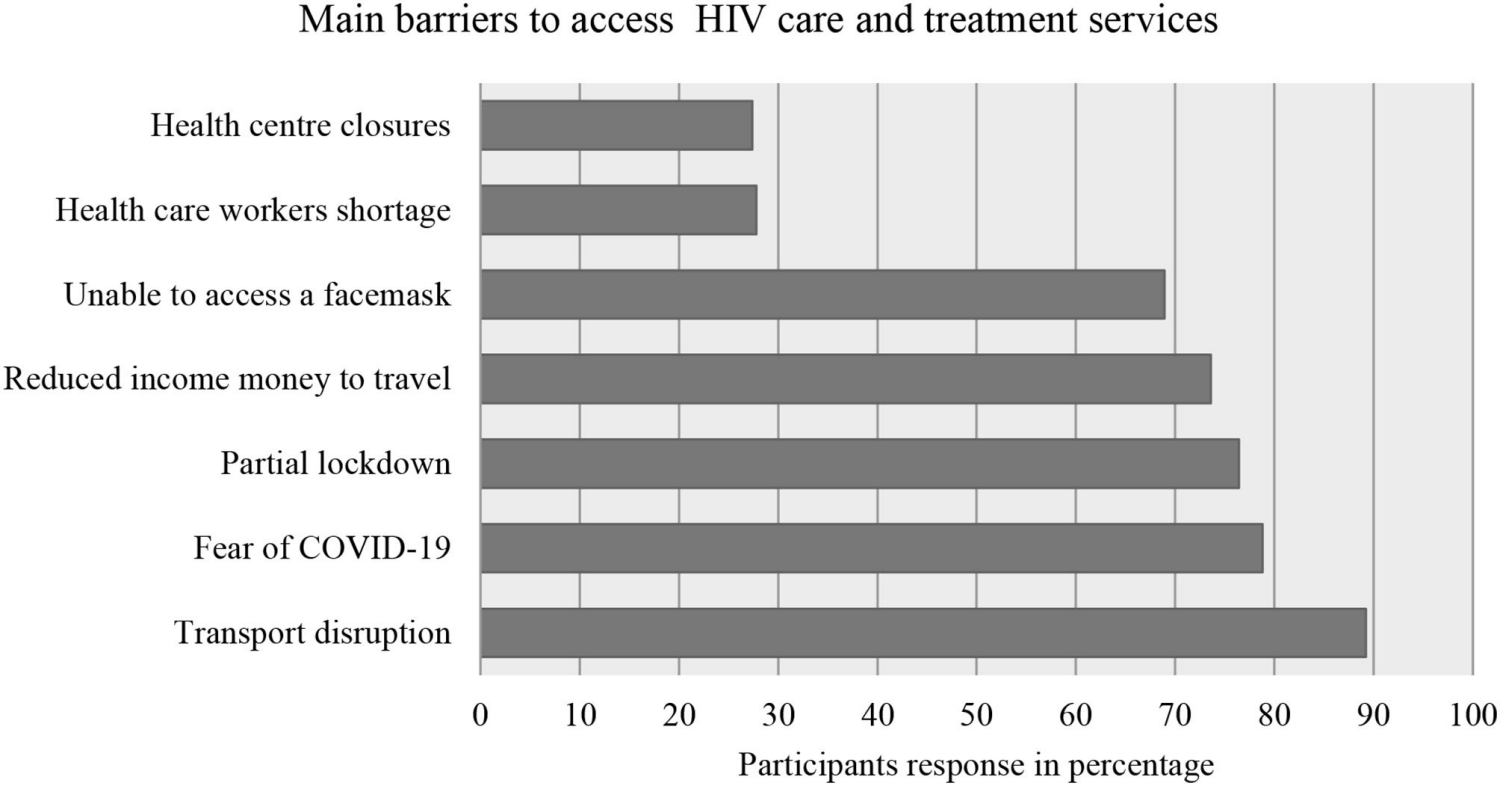
Response of the study participants regarding barriers in accessing health care and treatment during the pandemic, Addis Ababa, Ethiopia, May 2021.

Among the study subjects, 189 (89.2%) said transport disruption was the main barrier to access health care. Fear of getting infected with COVID-19 (78.8%) was the second main barrier for the participants (Figure 1).

### COVID-19 Precaution Measures in Healthcare Facilities

Among the participants, 143 (67.5%) responded that health centers provide screening services for COVID-19, and all health professionals wear masks. The participants responded that there were water (97.2%) and soap (95.8%) at the gate of the healthcare facilities, but not sanitizer (74.1%) (Table 6).

**Table 6:** Response of the study participants to the precautions of health facilities for COVID-19 protection, Addis Ababa, Ethiopia, May 2021.

**Variables**	**Category**	**Frequency**	**Percentage**
Health center provide screening service for COVID-19?	Yes	143	67.5%
	No	69	32.5%
Health professionals wear the gloves during caregiving?	Yes	211	99.5%
	No	1	0.5%
Health professionals wear the mask during caregiving?	Yes	212	100%
	No	0	0.0%
Water available at the entrance of the health center for hand washing?	Yes	206	97.2%
	No	6	2.8%
Soap available at the entrance of the health center for hand washing?	Yes	203	95.8%
	No	9	4.2%
Sanitizer available at the entrance of the healthcentre for cleaning of hands?	Yes	55	25.9%
	No	157	74.1%

### Medications and Follow-Ups During COVID-19

Among the total participants, 125 (59.%) said that the ordered drugs were available. Two hundred (94.3%) were able to collect their multi-month drug supply. The participants who missed appointments, follow-up tests, and counseling services were 58 (27.4%), 56 (26.4%), and 55 (25.9%), respectively (Table 7).

**Table 7:** Response of the study participants to medications and follow-up, Addis Ababa, Ethiopia May, 2021.

**Variables**	**Category**	**Frequency**	**Percentage**
Availability of ordered drugs?	Yes	125	59.0%
	Some	80	37.7%
	Not at all	7	3.3%
Non-medical support since COVID 19?	Same as before	163	76.9%
	Slightly harder	15	7.1%
	Much harder	23	10.8%
	Impossible	11	5.2%
Have you had multi-month drug supply	Yes	200	94.3%
	No	12	5.7%
For how many months	3 months	90	42.5%
	6 months	110	51.9%
Have you missed appointments (visits)	Yes	58	27.4%
	No	154	72.6%
Follow-up tests done	Yes	156	73.6%
	No	56	26.4%
Counseling done on your medication or healthstatus?	Yes	157	74.1%
	No	55	25.9%

### Logistic Regression Analysis of Missing Appointments/Visits for Medication Refill Variable

Bivariate and Multivariate Logistic Regression analysis showed that the following variables are significantly associated with the likelihood of missing appointments and medication refill. Independent variables, such as older age, less education, fear of COVID-19, transport disruption, reduced income, inability to access mask, no sanitizer availability, and high cost of disinfectant, were related to more missed appointments (Table 8).

**Table 8:** Bivariate and multivariate logistic regression analysis of missing appointments/visits for the medication refill variable, Addis Ababa, Ethiopia, 2021.

		**Missed appointments**		**Odds ratio**		
**Variables**	**Category**	**No**	**Yes**	**COR (CI)**	**AOR (CI)**	***P*-value**
Age	18–34 35–54 ≥55	43 (28.0%) 92 (59.7%) 19 (12.3%)	12 (20.7%) 11 (19.0%) 35 (60.3%)	1 0.43 (0.175–1.048) 6.60 (2.823–15.434)	1 0.41 (0.091–1.875) 6.73 (1.495–30.310)	0.252 0.013^*^
Education	No education Read + write Primary edu. Secondary edu. ≥ Diploma	14 (9.1%) 19 (12.3%) 50 (32.5%) 38 (24.7%) 33 (21.4%)	31 (53.5%) 11 (19.0%) 9 (15.5%) 6 (10.3%) 1 (1.7%)	1 0.25 (0.096–0.670) 0.08 (0.031–0.203) 0.07 (0.024–0.201) 0.01 (0.0012–1.021)	1 0.01 (0.001–0.165) 0.02 (0.002–0.229) 0.05 (0.003–1.022) 0.01 (0.001–1.002)	0.001^*^ 0.002^*^ 0.052 0.997
Fear of COVID-19	No Yes	44 (28.6%) 110 (71.4%)	1 (1.7%) 57 (98.3%)	1 22.80 (3.062–169.782)	1 24.93 (2.798–222.279)	0.004^*^
Transport disruption	No Yes	22 (14.3%) 132 (85.7%)	1 (1.7%) 57 (98.3%)	1 9.50 (1.250–31.185)	1 4.90 (1.031–23.174)	0.038^*^
Reduced income	No Yes	53 (34.4%) 101 (65.6%)	3 (5.2%) 55 (94.8%)	1 9.62 (2.873–32.219)	1 5.64 (1.234–25.812)	0.026^*^
Unable to access mask	No Yes	64 (41.6%) 90 (58.4%)	2 (3.4%) 56 (96.6%)	1 19.91 (4.687–84.577)	1 7.67 (1.303–45.174)	0.024^*^
Sanitizer available	No Yes	110 (71.4%) 44 (28.6%)	47 (81.0%) 11 (19.0%)	1 0.58 (0.278–1.231)	1 0.07 (0.007–0.729)	0.026^*^
For how many months	3 months 6 months	52 (35.9%) 93 (64.1%)	38 (69.1%) 17 (30.9%)	1 0.25 (0.129–0.486)	1 0.33 (0.132–0.825)	0.018^*^
Cost of disinfectant	No Yes	85 (55.2%) 69 (44.8%)	10 (17.2%) 48 (82.8%)	1 5.91 (2.788–12.539)	1 16.64 (1.462–189.569)	0.023^*^
Non-medical support since COVID-19	Same as before Slightly harder Much harder Impossible	130 (84.4%) 12 (7.8%) 10 (6.5%) 2 (1.3%)	33 (56.9%) 3 (5.2%) 13 (22.4%) 9 (15.5%)	1 0.98 (0.263–3.693) 5.12 (2.064–12.705) 17.72 (3.655–85.987)	1 3.68 (0.434–31.204) 3.78 0.774–18.421) 2.32 (1.547–12.596)	0.233 0.100 0.044^*^

* *Statistically significant at p < 0.05, COR, crude odds ratio at 95% confidence interval; AOR, adjusted odds ratio at 95% confidence interval*.

### Logistic Regression Analysis of the Follow-Up Tests Variable

In Bivariate and Multivariate Logistic Regression analysis of the follow-up tests variable, the following variables found to be significant: age, denied health services, reduced income/money to travel, partial lockdown, and inability to access face masks (Table 9).

**Table 9:** Bivariate and multivariate logistic regression analysis of the follow-up tests variable, Addis Ababa, Ethiopia, 2021.

		**Followup test**		**Odds ratio**		
**Variables**	**Category**	**No**	**Yes**	**COR (CI)**	**AOR (CI)**	***P*-value**
Age	18–34 35–54 ≥55	12 (21.4%) 10 (17.9%) 34 (60.7%)	43 (27.6%) 93 (59.6%) 20 (12.8%)	1 2.59 (1.041–6.472) 0.16 (0.070–0.382)	1 2.65 (0.913–7.670) 0.22 (0.076–0.621)	0.073 0.004^*^
Partial lockdown	No Yes	1 (1.8%) 55 (98.2%)	49 (31.4%) 107 (68.6%)	1 0.04 (0.005–0.295)	1 0.10 (0.011–0.833)	0.034^*^
Denied health services	No Yes	35 (62.5%) 21 (37.5%)	150 (96.2%) 6 (3.8%)	1 0.07 (0.025–0.177)	1 0.15 (0.045–0.475)	0.001^*^
Reduced income	No Yes	3 (5.4%) 53 (94.6%)	53 (34.0%) 103 (66.0%)	1 0.11 (0.033–0.369)	1 0.18 (0.039–0.784)	0.023^*^
Unable to get mask	No Yes	2 (3.6%) 54 (96.4%)	64 (41.0%) 92 (59.0%)	1 0.05 (0.013–0.226)	1 0.12 (0.026–0.543)	0.006^*^

* *Statistically significant at p < 0.05, COR, crude odds ratio at 95% confidence interval; AOR, adjusted odds ratio at 95% confidence interval*.

### Logistic Regression Analysis of the Counseling Variable

Bivariate and Multivariate Logistic Regression analysis of the counseling variable, factors such as age, education, fear of COVID-19, reduced income money to travel, inability to access face masks, and partial lockdown were significant (Table 10).

**Table 10:** Bivariate and multivariate logistic regression analysis of the counseling variable, Addis Ababa, Ethiopia, 2021.

		**Counslingdone**		**Odds ratio**		
**Variable**	**Category**	**No**	**Yes**	**COR (CI)**	**AOR (CI)**	***P*-value**
Age	18–34 35–54 ≥55	12 (21.8%) 10 (18.2%) 33 (60.0%)	43 (27.4%) 93 (59.2%) 21 (13.4%)	1 2.59 (1.041–6.472) 0.18 (0.077–0.412)	1 2.28 (0.842–6.170) 0.21 (0.078–0.570)	0.105 0.002^*^
Education	No education Read + write Primary edu. Secondary edu. ≥Diploma	29 (52.7%) 11 (20.0%) 8 (14.5%) 6 (10.9%) 1 (1.8%)	16 (10.2%) 19 (12.1%) 51 (32.5%) 38 (24.2%) 33 (21.0%)	1 3.24 (1.241–8.449) 11.95 (4.572–31.251) 11.87 (4.142–34.047) 4.60 (0.391–15.227)	1 3.68 (1.230–11.022) 11.46 (3.906–33.615) 6.48 (1.921–21.876) 1.23 (0.238–6.412)	0.020^*^ 0.000^*^ 0.003^*^ 0.801
Fear of COVID-19	No Yes	1 (1.8%) 54 (98.2%)	44 (28.0%) 113 (72.0%)	1 0.05 (0.006–0.354)	1 0.11 (0.013–0.912)	0.041^*^
Reduced income	No Yes	3 (5.5%) 52 (94.5%)	53 (33.8%) 104 (66.2%)	1 0.11 (0.034–0.380)	1 0.17 (0.041–0.699)	0.014^*^
Unable get face mask	No Yes	2 (3.6%) 53 (96.4%)	64 (40.8%) 93 (59.2%)	1 0.05 (0.013–0.233)	1 0.19 (0.039–0.959)	0.044^*^
Partial lockdown	No Yes	1 (1.8%) 54 (98.2%)	49 (31.2%) 108 (68.8%)	1 0.04 (0.005–0.304)	1 0.08 (0.008–0.790)	0.031^*^

* *Statistically significant at p < 0.05, COR, crude odds ratio at 95% confidence interval; AOR, adjusted odds ratio at 95% confidence interval*.

## Discussions

To the best of our knowledge, this study was the first of its kind to assess the impact of COVID-19 on HIV care and treatment services in Ethiopia. We studied the overlap between the two ongoing pandemics (HIV and COVID-19) in Ethiopia. The findings underscore several factors rendering HIV care and treatment services more difficult. A significant number of participants have missed appointments, follow-up tests, and counseling services due to COVID-19. The COVID-19 containment measures taken by the government, sociodemographic characteristics of the patients, and inconsistent access to personal protective equipment are the main factors that have hindered the retention and adherence of patients with HIV to their routine HIV care and treatment.

The patient living with HIV had great concerns about whether they are at high risk for the pandemic and the worse outcomes if they get infected with COVID-19. Research findings on these concerns have been in agreement with previous studies conducted elsewhere (12, 18, 19, 21, 22, 33, 34). Studies indicated that, although the pandemic affected the health care for all disease conditions, chronic patients such as people living with HIV are likely to be uniquely vulnerable (4, 17, 35). It has been reported that the elderly and the people with chronic conditions are more likely to be infected with COVID-19, and patients with HIV may miss appointments as a result. According to this study, older patients with HIV were more likely to miss the clinical care and treatment services given by the health centers. This finding is in agreement with the study done in Addis Ababa in Tikur Anbessa Specialized Hospital (TASH), Dessie town government, and private hospitals where older chronic patients were more likely to miss clinical appointments/visits (36, 37). The result of another study in Uganda was also consistent with this finding that older people were more likely to miss ART and related services (19, 38). In our findings, those who had formal education are more likely to have care and treatment services. This might be because the respondents who had formal education may have a deeper understanding of the negative consequence if they missed their follow-up visits and they could have more tendency to request and to access information about COVID-19 and its preventive measures.

Our results also indicated that patients with HIV, who had a fear of getting infected with COVID-19, were more likely to miss appointments for care and treatment. This is also consistent with other findings (39–41). Containment measures of COVID-19 taken in Ethiopia had a significant contribution to halting the spread of COVID-19 in Ethiopia; however, they had their own implications on HIV care and treatment services as the response from the patients with HIV as indicated. Transport disruption, partial lockdown that impaired mobility, and income reduction were significant factors in missing health care visits, which was in agreement with previous studies conducted in Ethiopia (13, 42), and elsewhere in the world (43–50) that the COVID-19 containment measure had a significant impact on patients' access to healthcare facilities.

Undue expenses related to protective equipment, including face masks and sanitizers, were a burden for the people living with HIV. This finding is in agreement with previous findings in Ethiopia (14) and elsewhere in Africa, wherein sufficient money to buy protective equipment was commonly reported effects of the COVID-19 on attending HIV clinical care and treatment services (51). The city of Addis Ababa introduced innovative measures providing ART medications for 3 to 6 months to mitigate these challenges. In our finding, those who collect medications for 6 months were less likely to miss appointments for medication refill compared to those who took for 3 months.

Indirect impacts arising from the pandemic, which reduced non-medical support, had economical burdens. The participants who said non-medical support was much harder and impossible were more likely to miss clinical visits. Similar observations were reported in other studies as well (52). Furthermore, WHO stated that the COVID-19 pandemic has affected the availability of medicines and non-medical supports in many countries as the world focused on the COVID-19 pandemic (53, 54). Indeed, health centers in Addis Ababa have had preeminent COVID-19 precaution procedures and measures to protect their clients from the pandemic. Availability of sanitizer, water, and soap at the health facilities' gates encouraged the patients with HIV to attend their routine care. These results are in line with a finding from North Shoa health care facilities, where patients who got sanitizer and other supports to protect themselves from the pandemic were more satisfied by health services and attended the clinical appointments/vests (14, 55).

Our study has some limitations. The study was limited to healthcare facilities in Addis Ababa, and, therefore, may not be representative of Ethiopia. As the study design was a cross-sectional study, it does not show a causal relationship and only provides a view of the impacts of COVID-19 in a specific period. Otherwise, the study was based on real-time, patient-level primary data, and it was conducted in a resource-constrained, high-HIV-burden country context.

## Conclusion

The COVID-19 had a significant burden on patients with HIV to attend their routine clinical care and treatment, which may lead to treatment failure and drug resistance. The impact was on their appointments for medication refills and clinical and laboratory follow-ups. Targeted initiatives are needed to sustain HIV clinical care and treatment services and improve the well-being of people living with HIV. Stakeholders, such as the Addis Ababa health bureau, the ministry of health, and others, should work in partnership to reduce the impact of this pandemic on those patients to maintain their economic well-being.

## Data Availability Statement

The raw data supporting the conclusions of this article will be made available by the authors, without undue reservation.

## Ethics Statement

The studies involving human participants were reviewed and approved by the Scientific and Ethics Review Committee of the Center for Innovative Drug Development and Therapeutic Trials for Africa (CDT-Africa), College of Health Sciences, Addis Ababa University. Ethical clearance and support letters were obtained from Addis Ababa public health research and emergency directorate, Addis Ababa City Government Health Bureau. The patients/participants provided their written informed consent to participate in this study.

## Author Contributions

DC collected the primary data, conducted the analyses, and drafted the manuscript. TM and YW contributed to the data collection and analysis and reviewed the manuscript. All the authors have read and approved the manuscript.

## Funding

This study was supported by the Center for Innovative Drug Development and Therapeutic Trials for Africa (CDT-Africa). TM was supported in part by the Fogarty International Center and National Institute of Allergy and Infectious Diseases of the US National Institutes of Health (D43TW009127).

## Author Disclaimer

The content is solely the responsibility of the authors and does not necessarily represent the official views of the CDT-Africa or the National Institutes of Health.

## Conflict of Interest

The authors declare that the research was conducted in the absence of any commercial or financial relationships that could be construed as a potential conflict of interest.

## Publisher's Note

All claims expressed in this article are solely those of the authors and do not necessarily represent those of their affiliated organizations, or those of the publisher, the editors and the reviewers. Any product that may be evaluated in this article, or claim that may be made by its manufacturer, is not guaranteed or endorsed by the publisher.

## Abbreviations

AAU: Addis Ababa University
COVID-19: Coronavirus Disease 2019
SARS-CoV-2: severe acute respiratory syndrome coronavirus 2
HIV: human immunodeficiency virus
PLWH: people living with HIV
HC: health center
ICU: intensive care unit
PPE: personal protective equipment
WHO: World Health Organization
AOR: adjusted odds ratio
CI: confidence interval.
